# High-fat diet increases gliosis and immediate early gene expression in *APOE3* mice, but not *APOE*4 mice

**DOI:** 10.1186/s12974-021-02256-2

**Published:** 2021-09-18

**Authors:** Nahdia S. Jones, Katarina Q. Watson, G. William Rebeck

**Affiliations:** Department of Neuroscience, Georgetown University, Washington, DC 20007 USA

**Keywords:** Obesity, High-fat diet, Apolipoprotein E, APOE, Inflammation, Gliosis

## Abstract

**Background:**

*APOE4* is the strongest genetic risk factor for Alzheimer’s disease (AD), and obesity is a strong environmental risk factor for AD. These factors result in multiple central nervous system (CNS) disturbances and significantly increase chances of AD. Since over 20% of the US population carry the *APOE4* allele and over 40% are obese, it is important to understand how these risk factors interact to affect neurons and glia in the CNS.

**Methods:**

We fed male and female *APOE3* and *APOE4* knock-in mice a high-fat diet (HFD-45% kcal fat) or a "control" diet (CD-10% kcal fat) for 12 weeks beginning at 6 months of age. At the end of the 12 weeks, brains were collected and analyzed for gliosis, neuroinflammatory genes, and neuronal integrity.

**Results:**

*APOE3* mice on HFD, but not *APOE4* mice, experienced increases in gliosis as measured by GFAP and Iba1 immunostaining. *APOE4* mice on HFD showed a stronger increase in the expression of Adora2a than *APOE3* mice. Finally, *APOE*3 mice on HFD, but not *APOE4* mice, also showed increased neuronal expression of immediate early genes cFos and Arc.

**Conclusions:**

These findings demonstrate that *APOE* genotype and obesity interact in their effects on important processes particularly related to inflammation and neuronal plasticity in the CNS. During the early stages of obesity, the *APOE3* genotype modulates a response to HFD while the *APOE4* genotype does not. This supports a model where early dysregulation of inflammation in *APOE4* brains could predispose to CNS damages from various insults and later result in the increased CNS damage normally associated with the *APOE4* genotype.

## Background

Apolipoprotein E (*APOE*) 4 is the strongest genetic risk factor for Alzheimer’s disease (AD) [47]. It is present in nearly 25% of the US population and over 50% of AD patients [51]. There are three different *APOE* alleles: *APOE2*, *APOE3*, and *APOE4*. These alleles encode single amino acid differences at position 112 or 158 [28]. *APOE*3 is the most common allele and thus *APOE3* homozygotes are defined as having an average risk of AD. Meanwhile, *APOE4* increases risk of AD: Heterozygous *APOE4* carriers are 3-4 times more likely to get AD, while *APOE4* homozygous individuals are 15 times more likely [28]. *APOE4* can increase the risk of cognitive deficits in healthy individuals and in *APOE4* knock-in mouse models [10]. Compared to homozygous *APOE3* mice, homozygous *APOE4* mice have increased cognitive deficits and decreased neuronal integrity without developing AD pathology [41]. *APOE4* mice crossed with mice that exhibit AD pathology have increased plaque accumulation and increased inflammation when compared to homozygous *APOE3* mice [42, 49].

Obesity is a strong environmental risk factor for AD [5, 35] and has been reported to affect over 40% of the US population [13]. Obesity is often accompanied by metabolic disturbances such as increases in adipose tissue, hyperglycemia, hypercholesterolemia, insulin resistance, and has increasingly been associated with cognitive deficits [13, 24]. Obesity in rodents can be modeled through high-fat diets (HFD) [25, 27]. Like in humans, rodents on HFD develop increased adipose tissue, glucose intolerance, and insulin resistance [21, 26]. In the central nervous system (CNS), HFD has been associated with increased inflammation (gliosis, cytokine expression) [40, 53] and behavioral deficits (Morris Water Maze), delayed matching, and non-matching task [7, 32].

Here, we investigate how diet induced obesity affects inflammation in the CNS in *APOE* mice. Since *APOE4* is the strongest genetic risk factor for AD and the prevalence of obesity is increasing, it is imperative to understand how the two risks interact and contribute to the eventual inflammatory state of AD. We previously found that HFD induced metabolic syndrome in both *APOE3* and *APOE4* mice with a stronger effect in *APOE4* mice [21]. Here, we have identified the effects of HFD on brain inflammation.

## Methods

### Animals/diet

Male and female *APOE3* and *APOE4* knock-in mice, expressing the human *APOE* allele in the mouse *APOE* gene locus, on a C57BL/6J background (the gift of Patrick Sullivan) were used. These mice model AD risk without pathological changes. They exhibit normal CNS expression of the *APOE* gene, but differ in gene expression profiles and behavior across the *APOE* genotypes [41, 48, 56]. They were fed either a HFD (45% kcal fat, Research Diets-D12451) or ingredient-matched "control" diet (CD) (10% kcal fat, Research Diets-D12450H) for 12 weeks beginning at 6 months of age [21]. HFD fat content was predominantly lard-based, making it high in omega-6 fatty acids (ingredients: 177 g lard, 25 g soybean oil in 858 g of diet), while CD fat content was predominantly soybean oil based making it higher in omega-3 fatty acids (ingredients: 20 g lard, 25 g soybean oil in 1055 g of diet). Food and water were provided ad libitum. At the end of the 12 weeks, mice were euthanized and brains collected for analysis. Mice were euthanized by CO_2_ inhalation and brains perfused with 1x PBS. Brains of *APOE3* and *APOE4* mice on CD and HFD (*n* = 13-15 per genotype and diet, 5-9 per sex) were extracted, and hemi-sections were divided for immunofluorescence (IF) assays, Golgi staining, and biochemical assays. All experiments followed the guidelines of the Georgetown University Institutional Animal Care and Use Committee.

### Immunofluorescence

**Fig. 1:**
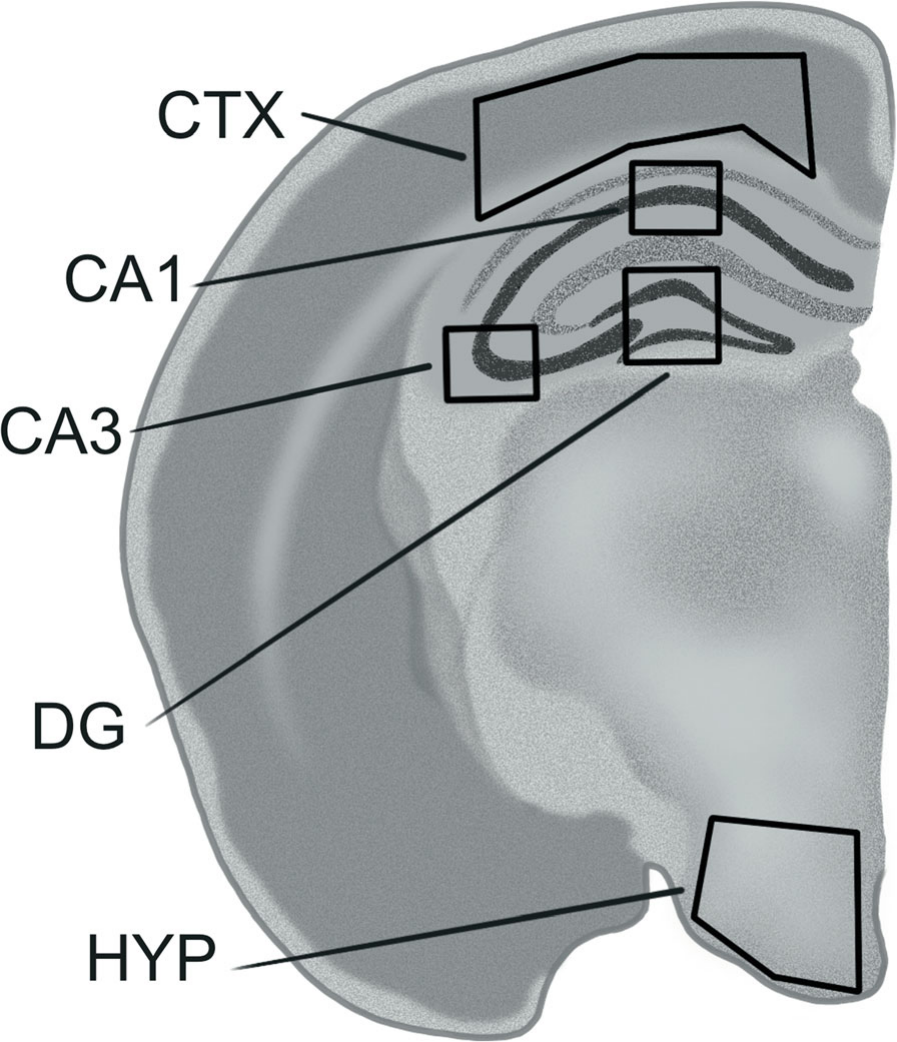
Illustration of mouse brain hemisphere with areas analyzed for IF outlined

For IF (*N* = 3-4 per genotype and diet, 1-2 per sex), one brain hemisphere was fixed in 4% PFA and sucrose before freezing in cold 2-methylbutene and slicing at 30 μm. IF stains were performed for Iba1 (Wako, Cat #: 019-19741), GFAP (Cell Signaling, Cat #: 3670S), Doublecortin (DCX, ThermoFisher, Cat #: 481200), NeuN (Chemicon, Cat #: MAB377), and cFOS (Abcam, Cat # ab190289). Stains were imaged using a Zeiss AxioSkop at either 10× (Iba1, GFAP, and cFOS) or 20× (co-stains and DCX) magnification. Staining was analyzed using Image J, with quantification from 4 brain slices/mouse. Hippocampal images were taken from the stratum radiatum and stratum oriens of CA1 and CA3, and the CA4 and the molecular layers (MO) of the dentate gyrus (DG). They were also taken from the arcuate nucleus, the dorsal medial, and lateral hypothalamus, and from multiple layers of the cerebral cortex (Fig. 1). Iba1 and GFAP were analyzed as the percent area covered by IF staining in the image. DCX and cFos expression were analyzed as the counts of antibody positive cells. All quantification was conducted blinded.

### Golgi staining

Brain hemisections were placed in Golgi staining solutions according to recommendations (FD Rapid GolgiStain Kit (PK401)) and sliced at 150 μm. Images of the basal shaft (BS) and apical oblique (AO) dendrites of neurons in the entorhinal cortex were acquired with an Olympus XB51 microscope at 60× magnification with oil immersion. A minimum of 10 neurons were imaged for each mouse (*N* = 4-5 mice per genotype and diet, 2-3 per sex). Dendritic lengths were measured, and spines counted using Image J. All quantification was conducted in a blinded manner.

### RNA analyses

Cortex from one brain hemisphere (*N* = 5-7 per genotype and diet, 2-4 per sex) was used for RNA isolation. RNA was isolated using the Direct-zol RNA Miniprep kit (Zymo Research R2051) and analyzed by Georgetown University’s Genomic and Epigenomics Shared Resource to determine concentration and purity. For preliminary studies of brain inflammation, equal amounts of four samples for each condition were pooled and analyzed on the NanoString nCounter Mouse Neuroinflammation Panel (Cat #XT-CSO-MNROI1, *N* = 4 pooled per genotype and diet), which measures transcripts of 770 neuroinflammatory genes. For studies of brain metabolism, three independent samples from each condition were analyzed on the NanoString nCounter Mouse Metabolic Pathways Panel (Cat #XT-CSO-MMP1-12, *N* = 3 per genotype and diet), which detects transcripts of 768 metabolic genes. All data were analyzed using nSolver 4.0. Background was subtracted from negative controls and samples were normalized to the positive controls and housekeeping genes. Data were further analyzed by normalized count and fold difference, and differences were compared across diet and genotype.

mRNA species that demonstrated at least a twofold difference were subsequently analyzed using qRT-PCR. cDNA (*N* = 5-7 per genotype and diet, 2-4 per sex) was produced from extracted RNA using the High-Capacity cDNA Reverse Transcription Kit (ThermoFisher Cat #: 4368814). qRT-PCR for Adora2a, Arc, cFOS, C3, Egr1, IL-3, IL-6, and TNFα were run with the sequences and temperatures described in Table 1. All qRT-PCR were performed in triplicates using the Power SybrGreen Mix (Applied Biosystems) and a 7900HT fast qRT-PCR system. Results were normalized to housekeeping gene GAPDH. Data were analyzed as fold difference compared to CD *APOE3* samples as described [29].

**Table 1:** Primer sequences for qRT-PCR

Primer	Sequence	Temperature
Adora2a	Fwd: AGCAACCTGCAGAACGTCACAAAC Rev: TGGCAATAGCCAAGAGGCTGAAGA	60
Arc	Fwd: GGAGGGAGGTCTTCTACCGTC Rev: CCCCCACACCTACAGAGACA	57.5
C3	Fwd: GACGCCACTATGTCCATCCT Rev: CCAGCAGTTCCAGGTCCTTTG	57.5
cFos	Fwd: CTCTGGGAAGCCAAGGTC Rev: CGAAGGGAACGGAATAAG	55
Egr1	Fwd: GAGGAGTTATCCCAGCCAA Rev: GGCAGAGGAAGACGATGAAG	57.5
GAPDH	Fwd: GTGTTTCCTCGTCCCGTAGA Rev: ATTCCGTTCACACCGACCTT	55, 57.5, 60
IL3	Fwd: CCTGGGACTCCAAGCTTCAA Rev: GACAATAGAGCTGCAATTCAACGT	57.5
Il6	Fwd: ACGGCCTTCCCTACTTCACA Rev: CATTTCCACGATTTCCCAGA	57.5
TNF-a	Fwd: GGTGCCTATGTCTCAGCCTCTT Rev: GCCATAGAACTGATGAGAGGGAG	60

### Statistical analyses

All data are expressed as mean ± standard deviation. Comparisons among *APOE* genotypes and diets were analyzed by two-way ANOVAs with Sidak’s multiple comparison test. Statistical significance was determined by a probability error of *p* < 0.05. Sample sizes were included throughout the “Methods” section for each assay. All analyses and graph plotting were done using GraphPad Prism 8.

## Results

### HFD increases Iba1 immunoreactivity in *APOE3* and *APOE4* mice

**Fig. 2:**
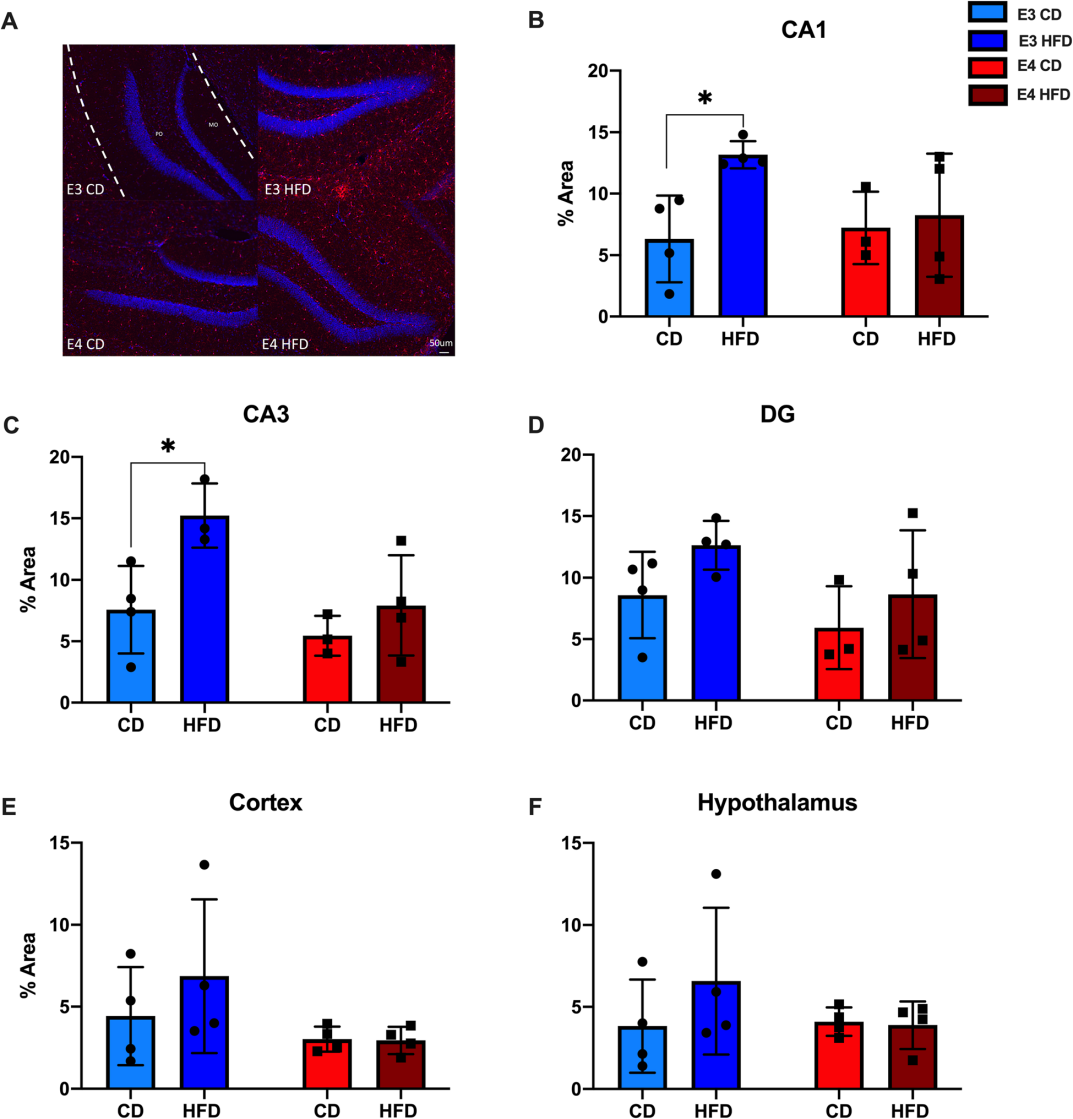
HFD *APOE3* mice, but not *APOE4* mice show increased Iba1 immunoreactivity. **A** Images of Iba1 immunoreactivity in DG of the HPC. **B**-**F** Comparison of HFD effects on Iba1 immunoreactivity in CA1, CA3, and DG of the HPC, the CTX, and HYP. E3 CD: light blue, *APOE3* mice on a CD, E3 HFD: dark blue, *APOE3* mice on a HFD, E4 CD: light red, *APOE4* mice on a CD, E4 HFD: dark red. *N* = 3-4, two-way ANOVA, *p < 0.05

To examine whether HFD increased microglial activation, we measured Iba1 immunoreactivity in the hippocampus (HPC), cortex (CTX), and hypothalamus (HYP) of *APOE3* and *APOE4* mice either fed HFD or CD (Fig. 2A). In CA1 of the HPC, HFD *APOE3* mice had significantly more Iba1 immunoreactivity when compared to CD *APOE3* mice (Fig. 2B, *p* = 0.035). There were no significant differences between HFD and CD *APOE4* mice. Similarly, in CA3 of the HPC, HFD *APOE3* mice had significantly more Iba1 immunoreactivity when compared to CD *APOE3* mice (Fig. 2C, *p* = 0.024) and there were no significant differences between HFD and CD *APOE*4 mice. In the dentate gyrus (DG) of the HPC, there were no significant differences among the groups, although the pattern mirrored CA1 and CA3 results (Fig. 2D). In the CTX and HYP, neither HFD *APOE3* nor HFD *APOE4* mice had significant differences in Iba1 immunoreactivity when compared to CD mice (Fig. 2E-F). For all areas, there were no statistically significant differences between HFD *APOE3* and HFD *APOE4* mice or between CD *APOE3* and CD *APOE4* mice.

### HFD increases GFAP immunoreactivity in *APOE3* and *APOE4* mice

**Fig. 3:**
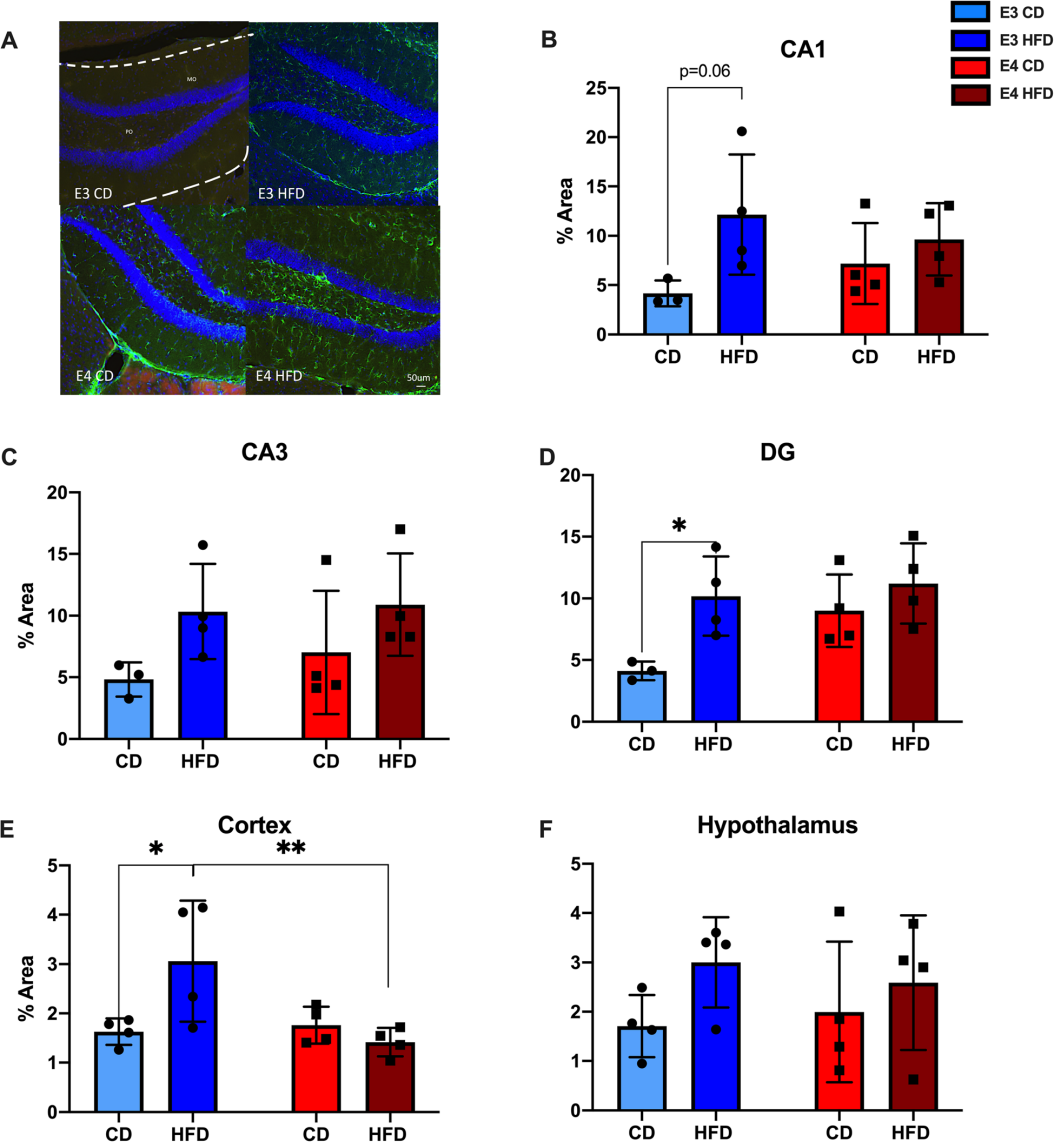
HFD *APOE3* mice, but not *APOE4* mice show increased GFAP immunoreactivity. **A** Images of GFAP immunoreactivity in DG of the HPC. **B**-**F** Comparison of HFD effects on GFAP immunoreactivity in CA1, CA3, and DG of the HPC, the CTX, and HYP. *N* = 3-4, two-way ANOVA, *p < 0.05, **p < 0.005

To examine whether HFD increased astrocytic activation, we analyzed GFAP immunoreactivity in the same brain regions (Fig. 3A). In CA1 of the HPC, HFD *APOE3* mice trended toward significantly more GFAP immunoreactivity than CD *APOE3* mice (Fig. 3B, *p* = 0.067); there was no difference between HFD *APOE4* and CD *APOE4* mice. In CA3 of the HPC, neither HFD *APOE3* nor HFD *APOE4* mice had significantly more GFAP immunoreactivity when compared to CD mice (Fig. 3C). In the DG of the HPC, HFD *APOE3* mice had significantly more GFAP immunoreactivity when compared to CD *APOE3* mice (Fig. 3D, *p* = 0.035), and there was no significant difference between HFD *APOE4* and CD *APOE4* mice. In the CTX, HFD *APOE3* mice also had significantly more GFAP immunoreactivity than CD *APOE3* mice (Fig. 3E, *p* = 0.02); again, there was no difference between HFD *APOE4* and CD *APOE4* mice. In the HYP, there were no significant differences by diet or genotype (Fig. 3F). When compared across genotypes, there were no differences except for in the CTX where HFD *APOE3* mice had significantly more GFAP immunoreactivity than HFD *APOE4* mice (Fig. 3E, *p* = 0.009). Thus, HFD was associated with higher measures of gliosis (Iba1 and GFAP) across brain regions in *APOE3* mice, but not in *APOE4* mice.

### HFD increases immediate early gene expression in *APOE3*, but not *APOE4* mice

To identify genes related to neuroinflammation or metabolism that may be related to HFD and *APOE*, we used a NanoString neuroinflammatory panel and a NanoString metabolic panel, and assessed over 1400 genes involved in neuroinflammation and metabolism. The panels included categories such as adaptive immune response, apoptosis, astrocyte function, inflammatory signaling, innate immune response, lipid metabolism, glucose transport, glycolysis, and fatty acid oxidation and synthesis. The panels also included *APOE* expression, which did not significantly differ by diet or genotype. From these categories, the two genes that had the highest expression, indicated by the neuroinflammatory panel, and that showed more than a twofold change after HFD were two immediate early gene (IEGs), cFos and Arc. IEGs can be altered by chronic activation of the innate immune system; under inflammatory conditions, increased IEG expression has also been associated with glia-induced increases in neuronal activity [43].

**Fig. 4:**
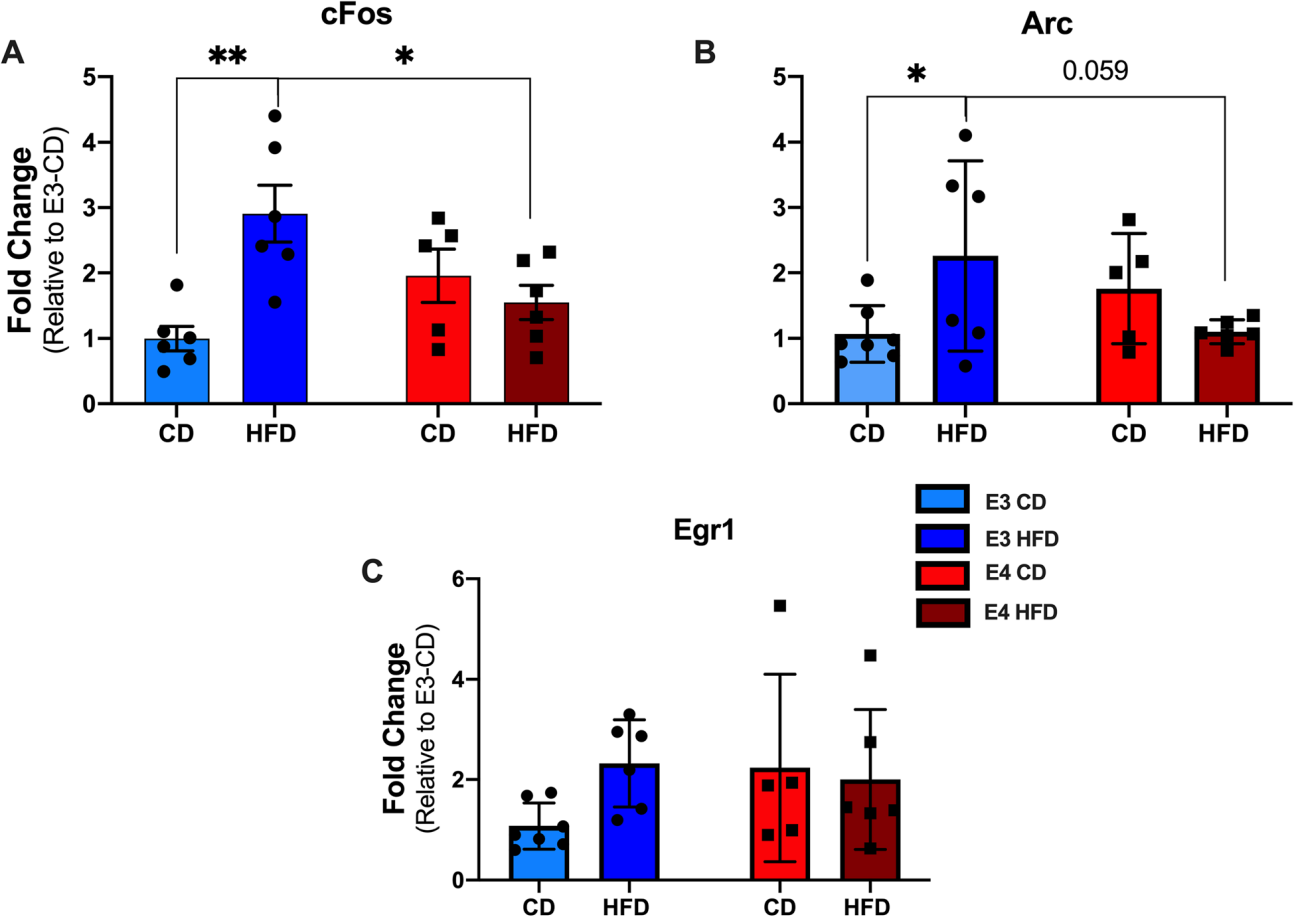
HFD *APOE3* mice, but not *APOE4* mice have increased IEG expression. **A** Comparison on HFD effects on fold changes in cFOS gene expression. **B** Comparison on HFD effects on fold changes in Arc gene expression. **C** Comparison on HFD effects on fold changes in Egr1 gene expression. *N* = 5-6, two-way ANOVA, *p < 0.05, **p < 0.005

To examine IEG expression across individual brain samples, we used qRT-PCR. Consistent with the pilot data, HFD *APOE3* mice had significantly higher cFos gene expression than CD *APOE3* mice (Fig. 4A, 66%, *p* = 0.001), while there was no significant difference between HFD *APOE4* and CD *APOE4* mice. When compared across *APOE* genotypes, there were no differences in expression between CD *APOE3* mice and CD *APOE4* mice, but HFD *APOE3* mice had significantly higher gene expression than HFD *APOE4* mice (Fig. 4A, *p* = 0.013).

Similar to the pattern of expression of cFos, Arc levels were significantly higher in HFD *APOE3* mice compared to CD *APOE3* mice (Fig. 4B, 55%, *p* = 0.04); again, there was no significant difference between HFD *APOE4* and CD *APOE4* mice. When compared across *APOE* genotypes, HFD *APOE3* mice trended toward higher gene expression than HFD *APOE4* mice (Fig. 4B, *p* = 0.059), and there were no differences in expression between CD *APOE3* mice and CD *APOE4* mice. To further test the effects of diet on IEG, we analyzed Erg1, another IEG involved in learning and memory that was not represented on the neuroinflammation panel. Erg1 did not show significant differences between diet or genotype, although its pattern of expression did mirror cFOS and Arc (Fig. 4C).

**Fig. 5:**
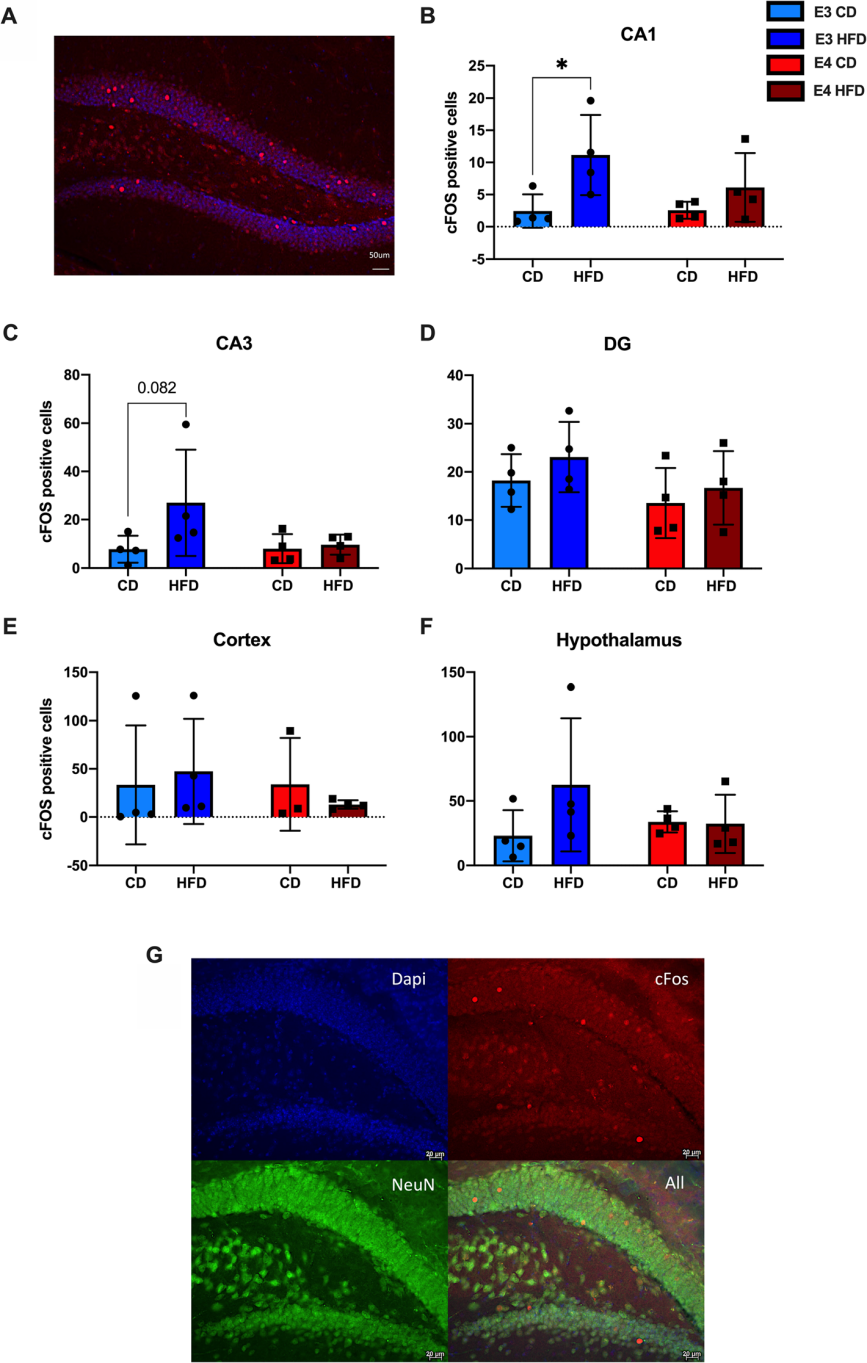
HFD *APOE3* mice, but not *APOE4* mice have increased cFOS immunoreactivity. **A** Image of cFOS positive cells in the DG. **B**-**F** Quantification of cFOS positive cells in CA1, CA3, and DG of the HPC, the CTX, and HYP. **G** Representative image of cFOS positive cells colocalized with neurons. *N* = 3-4, two-way ANOVA, *p < 0.05

To analyze IEG protein in the brain across *APOE* genotypes and diet, we used IF for cFOS and quantified cFOS positive cells in the HPC, CTX, and HYP (Fig. 5A). In CA1 of the HPC, HFD *APOE3* mice showed increased cFOS-positive cells when compared to CD *APOE3* mice (Fig. 5B, *p* = 0.03), consistent with the mRNA data. There was no difference between HFD *APOE4* and CD *APOE4* mice and no differences when compared across genotypes. In CA3, HFD *APOE3* mice trended toward more cFOS positive cells when compared to CD *APOE3* mice (Fig. 5C, *p* = 0.082). In the DG, there were no significant differences, although the expression mirrored the findings in CA1 and CA3 (Fig. 5D). There were no significant differences in the CTX or HYP (Fig. 5E-F). Since cFOS can be present in either glia or neurons [2], we co-stained for cFOS and NeuN to test whether the cFOS was colocalized to neurons. All cFOS-positive cells co-stained for NeuN, across genotypes and diets (Fig. 5G).

### HFD differentially affects other neuroinflammatory genes in *APOE3* and *APOE4* mice

**Fig. 6:**
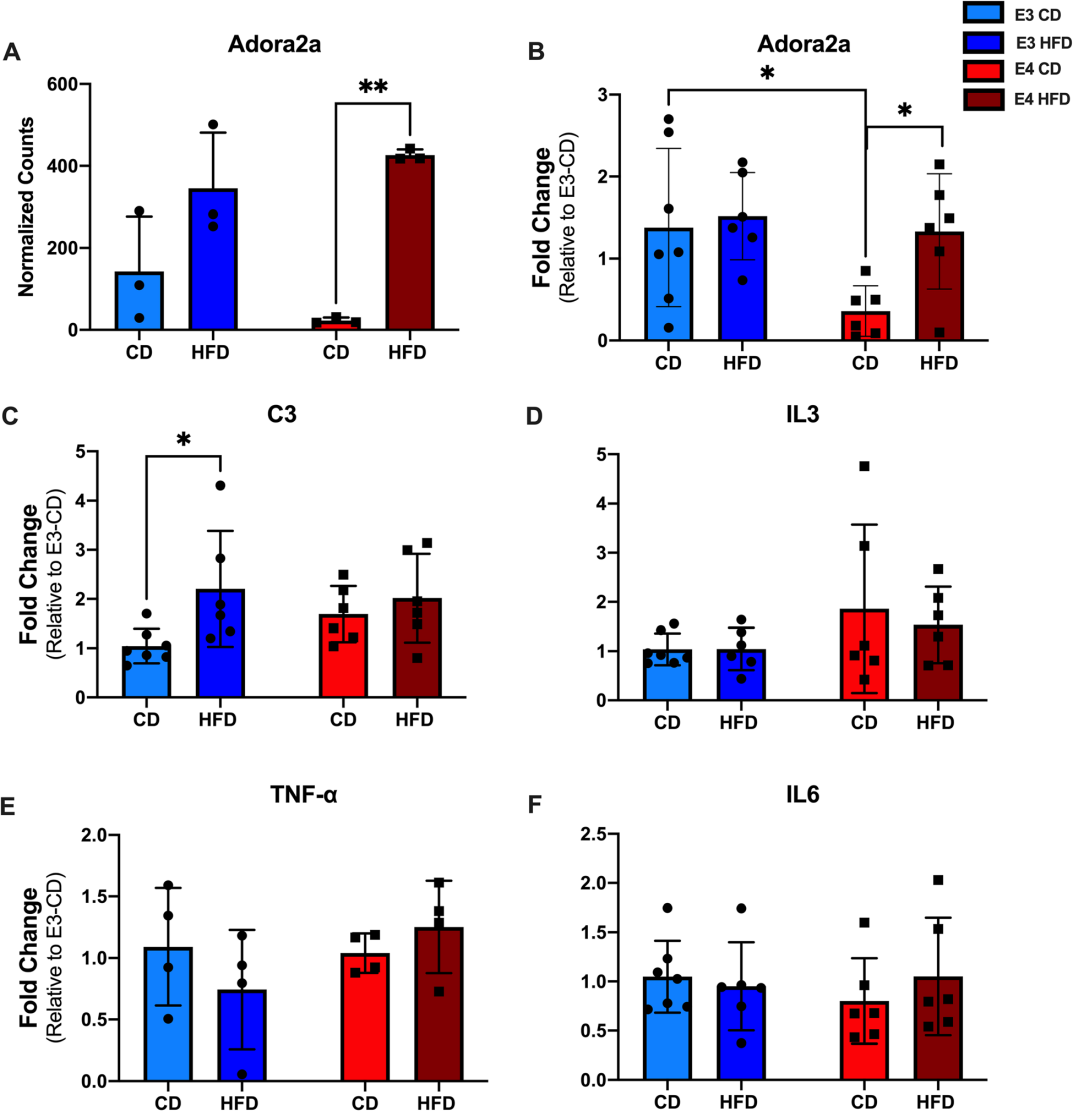
HFD alters specific neuroinflammatory genes. **A** Comparison on HFD effects on NanoString nCount of Adora2a gene expression. **B** Comparison on HFD effects on fold changes in Adora2a gene expression. **C** Comparison on HFD effects on fold changes in C3 gene expression. **D** Comparison on HFD effects on fold changes in IL-3 gene expression. **E** Comparison on HFD effects on fold changes in TNF-a gene expression. **F** Comparison on HFD effects on fold changes in IL-6 gene expression. **A** *N* = 3, two-way ANOVA, *p < 0.003. **B**-**F** *N* = 4-6, two-way ANOVA, *p < 0.05

Along with IEGs, the NanoString nCounter mRNA analysis panel indicated changes in several other genes related to neuroinflammation: Adora2a, C3, and IL-3. Adora2a is an anti-inflammatory gene with properties protective against tissue damage [37, 50]. HFD *APOE4* mice exhibited significantly higher levels of Adora2a gene expression than CD *APOE4* mice both by NanoString nCounter analysis (Fig. 6A, *p* = 0.002) and qRT-PCR analysis (Fig. 6B, *p* = 0.046). There were no differences between HFD and CD *APOE3* mice. When compared across *APOE* genotypes, there were no significant differences in Adora2a expression between mice with the NanoString nCounter analysis, but with qRT-PCR, CD *APOE3* mice had significantly higher gene expression than CD *APOE4* mice (Fig. 6B, *p* = 0.029). There were no differences between HFD *APOE3* and HFD *APOE4* mice. Thus, HFD increased Adora2a expression, particularly in the *APOE4* mice.

The remaining genes indicated by the neuroinflammatory panel were only analyzed by qRT-PCR because of the lower mRNA expression levels. The complement factor C3 plays an important role in innate immunity. HFD *APOE3* mice exhibited significantly higher levels of C3 gene expression than CD *APOE3* mice (Fig. 6C, *p* = 0.032). There were no differences between HFD and CD *APOE4* mice and no differences by *APOE* genotype. To examine C3 activity, we ran a western blot and examined C3 and cleaved C3; however, we did not find any difference in C3 or cleaved C3 levels (data not shown). For the other neuroinflammatory gene identified (IL-3), as well as the common markers of general inflammation TNF-α and IL-6, there were no differences by diet or genotype (Fig. 6D-F).

### HFD does not alter neurogenesis or spine density in *APOE3* and *APOE4* mice

**Fig. 7:**
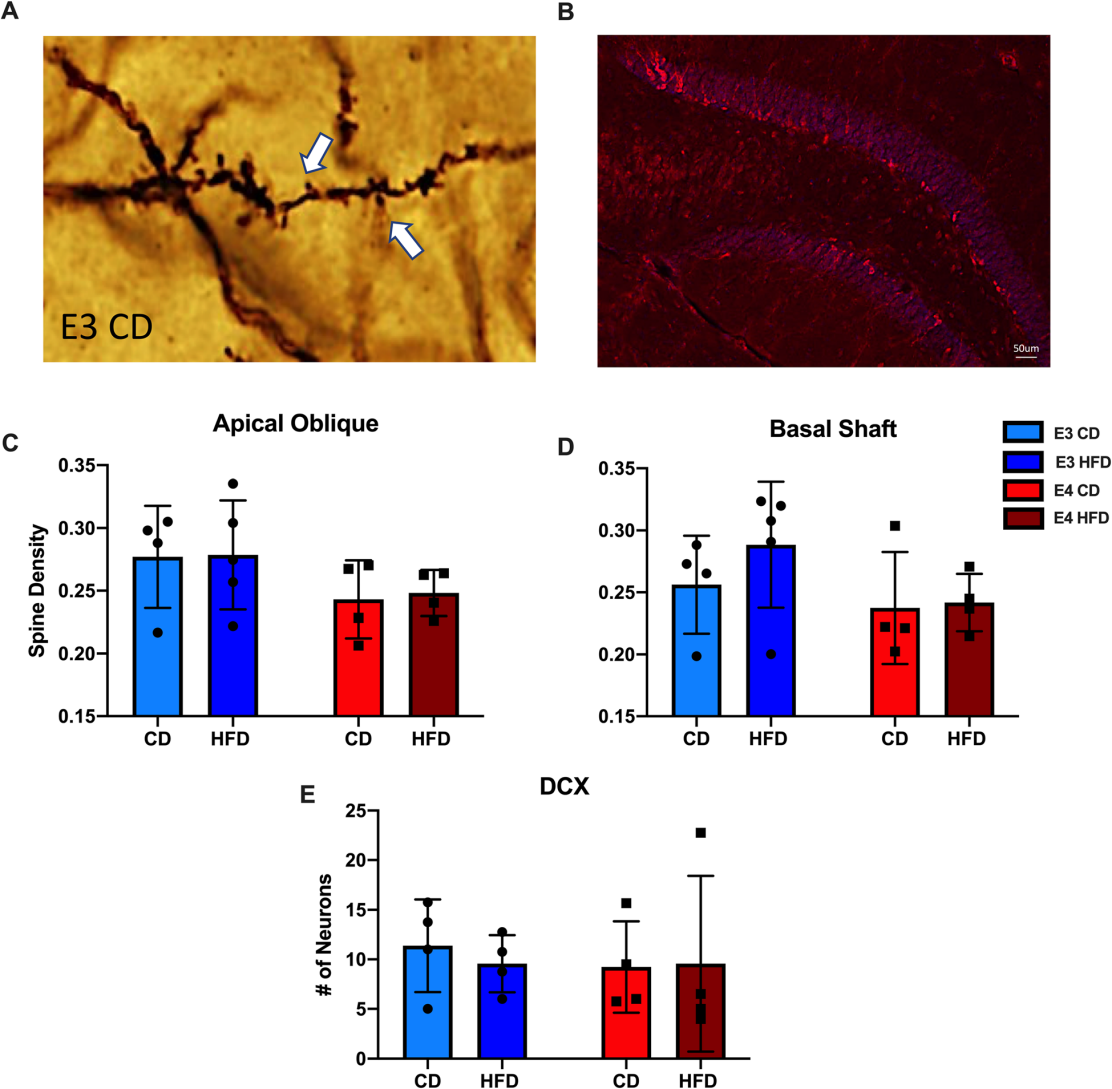
HFD does not alter neurogenesis or spine density in *APOE3* and *APOE4* mice. **A** Image of spines quantified from Golgi staining. **B** Image of DCX positive cells. **C**-**D** Quantification of spine density on the Apical Oblique and Basal Shaft. **E** Quantification of DCX positive cells

Neuronal degeneration has been associated with obesity and inflammation [3]. To test whether HFD here affected neuronal integrity, we examined spine density and hippocampal neurogenesis (Fig. 7A-B). For spine density, Golgi-stained pyramidal neurons in the entorhinal cortex were analyzed for dendritic spine density, quantified on the apical oblique and basal shaft dendrites. There were no significant differences in spine density across genotypes or diets (Fig. 7C-D). To examine whether HFD affects neurogenesis, we analyzed neurons in the DG of the HPC by DCX immunostaining. There were no significant differences in neurogenesis across genotypes or diets (Fig. 7E).

## Discussion

It is important to understand how environmental factors combine with genetic factors to affect inflammation and alter the risk of AD. Here, we focused on the rapidly growing environmental risk factor, obesity, and the strongest genetic risk factor, *APOE4*, and sought to understand how the combination affects inflammation. Using a mouse model of the human *APOE* alleles, we found that HFD induced increased microglia and astrocyte expression in *APOE3*, but not *APOE4* brains. This same pattern continued with increases in the IEGs cFOS and Arc in *APOE3*, but not *APOE4* brains. These *APOE*-related CNS effects occur along with multiple metabolic disturbances: weight gain, adipose tissue accumulation, and glucose intolerance. These peripheral changes occurred predominantly in *APOE4* mice; no behavioral changes were seen as a result of the HFD [21].

Previously HFD has been associated with increases in both microglial and astrocytic activity in wild-type mice [40, 53]. The *APOE* mice in our study are similar to control mice in that they do not have accumulations of Aβ or phospho-tau. Furthermore, the *APOE3* mice act as controls and perform similarly to wild-type mice. Consistent with the study of wild-type mice, *APOE3* mice in our study showed an increase in microgliosis and astrocytosis, although *APOE4* mice did not. Another study showed HFD caused a decrease in CD68 (a marker of monocyte-derived cells) in *APOE4* mice [17]. While data support that *APOE4* mice have an increased response to short-term noxious stimuli such as LPS treatment or injury [23, 52, 57], there is evidence of a decreased response to the chronic inflammation caused by obesity [8, 17]. In one study, *APOE3* and *APOE4* mice were fed a HFD for approximately 7 months, and there were *APOE3*-specific increases in expression of TNFα, IL-6, and CD36 when compared to *APOE4* mice [8]. *APOE4* mice also exhibited lower expression of LPS immune sensors indicating a decreased ability for innate immune detection [8]. We propose that *APOE3* mice have wild-type responses to chronic inflammatory conditions, while *APOE4* mice may more readily respond to specific acute stimuli but have a more muted response to chronic stimuli.

Several studies have addressed the effects of HFD in AD mouse models related to *APOE*. HFD increases both Aß and phospho-tau pathology across AD mouse models [22, 27]; similar effects are seen in AD-*APOE* mouse models, but the responses differ by *APOE* genotype. In EFAD mice (mice that possess both a human *APOE* genotype and 5xFAD transgenes) on a HFD, there was an increase in gliosis and AD pathology in E4FAD mice, but not in E3FAD mice [34]. In APP/*APOE* mice (*APOE* mice crossed with APP/PS1ΔE9), there was an increase in AD pathology in *APOE4*, but not *APOE3* mice on HFD [36]. The HFD may be affecting an already compromised inflammatory system responding to the increased amyloid found in the *APOE4* mice.

We propose that, while chronic inflammation is damaging, inflammation at an early stage of obesity may be neuroprotective, and generally not experienced in *APOE4* mice. Inflammation is associated with neuroprotection in early disease stages [15, 45] and the response in the *APOE3* mice could be indicative of a similar mechanism. In AD mouse models, *APOE3* has been associated with increases in microglial-plaque interactions when compared to *APOE4* mice [46], an example of a positive inflammatory response in the *APOE3* CNS (although there might be differences based on plaque morphology [42]). The inflammatory response from *APOE3* mice can be beneficial for Aß clearance and, without it, the *APOE4* mice may suffer.

In addition to inflammatory processes, glia are also heavily involved in lipid metabolism. Increased gliosis in the *APOE3* mice may be a response to increased lipids and processing of lipids. Fatty acids can cross the blood-brain barrier and be taken up and stored by astrocytes; this ability prevents lipid induced neuronal damage [4]. Microglia also respond to increases in lipids and, when healthy, assist in the clearance of excess lipids [30]. The measures of astrogliosis and microgliosis in *APOE3* brains could reflect changes to increased CNS lipids, and the *APOE4* mice may have a deficit in responding to lipid-related stressors.

One gene significantly altered by diet and *APOE* genotype in the NanoString metabolic panel was Adora2a (also known as the adenosine A_2A_ receptor), which is associated with inflammation [11]. While the panel had multiple metabolic categories, none of the genes specifically associated with metabolism were altered. Adora2a is expressed in the cortex and hippocampus; after CNS damage, there is an increase of Adora2a expression on glia, which is associated with increased activity and inflammation [6]. In multiple neurodegenerative conditions, there is an increase in Adora2a resulting in neuronal excitotoxicity and cell death [6]. Here, we see an increase in Adora2a in *APOE4* mice on HFD, indicating potential enhancement in glutamatergic activity [11], which could lead to neuronal damage if prolonged. While the *APOE4* mice do not demonstrate increased gliosis at this stage of developing obesity, there could be stronger inflammation and neuronal damage if increased Adora2a expression persisted.

An *APOE3*-specific response to HFD was further emphasized through expression of IEGs indicated by the NanoString neuroinflammatory panel and confirmed by qRT-PCR. We found that HFD increased cFOS and Arc expression twofold in *APOE3* mice, with no effects in *APOE4* mice. This expression was observed in neurons, with the counts of cFOS-positive neurons reproducing the effects of HFD on mRNA measures. IEGs are genes that are often rapidly and transiently expressed as direct responses to stimuli such as novel environments or injury. IEG expression is also associated with alterations in synaptic plasticity, with either up- or downregulation of IEGs being associated with increased and decreased LTP and memory retention, respectively [1, 12, 33]. Changes in IEG activity are also associated with behavioral tasks such as novel object recognition, and fear conditioning, indicative of their role in learning [33].

IEG expression also changes from neuronal stress or damage. IEG expression is increased in AD brain [31]. These increases have been linked to more GFAP positive astrocytes and thioflavin-stained plaques [2] and in Aβ-mediated apoptosis [31]. There are also alterations in neuronal IEG expression after traumatic brain injuries [14, 38] and after chronic LPS-induced inflammation [43, 44]. After chronic LPS infusion, mice were tested on a memory paradigm and those treated with LPS had increased IEG expression. This increased IEG expression was directly correlated with increased microglia activity [44]. Overall, the chronic inflammation led to gliosis and that resulted in increased IEG activity and cognitive deficits [44]. We propose a similar process in our study, with HFD, in the *APOE3* but not *APOE4* mice, increasing gliosis and that gliosis is leading to altered IEG expression.

In many of the published studies, IEG expression was measured after behavioral stimulations such as learning and memory challenges. Here, we have not directly induced IEG expression, suggesting another mechanism. There may be *APOE3* specific neuronal adaptations to HFD expressed through IEG activity. IEG increases have been noted without additional stimuli in the HYP after an extended period on HFD, resulting in adipose tissue storage [54]. IEG activity also acts as an indicator of neuronal activity resulting in long-term adaptations within neural circuits [55]. The increased IEG expression we are seeing could affect the processing and storage of specific lipid components of the HFD, and that could lead to increased protection in *APOE3* mice. Lack of HFD-induced IEG activity, as in the *APOE4* mice, could lead to the more detrimental outcomes. The NanoString neuroinflammatory panel only indicated robust changes in the IEGs in *APOE3* mice on HFD. There was no indication of more severe neuroinflammation, further emphasizing that the mice are not currently suffering from detrimental neuroinflammation. Rather, the *APOE3* mice may be exhibiting compensational neuronal adaptations in response to HFD.

The *APOE3* mice exhibited several CNS alterations associated with HFD that the *APOE4* mice did not. These *APOE3* mice also showed decreased metabolic disturbances compared to *APOE4* mice [21]. We propose that during HFD, *APOE3* mice are able to induce protective inflammatory pathways that have positive peripheral and CNS effects. These mice were 10 months old at the end of all experimentation and were on HFD for a total of 4 months; therefore, we may be seeing the preliminary effects of HFD as they continue to gain weight. Prolonged obesity could result in more detrimental effects such as increased noxious inflammation, neuronal damage, and behavioral deficits. *APOE4* mice on HFD for extended periods or on diets with higher fat contents exhibit increased inflammation and behavioral deficits [18–20].

The *APOE4* allele is the ancestorial version with the *APOE3* allele potentially evolving as a result in shifts in diet availability [9, 16, 39]. Evolutionarily, *APOE3* may allow humans to compensate for increased diet availability more easily and to process dietary fats, while *APOE4* remains better suited for environments where diet availability is scarce.

## Conclusion

Overall, we observed increases in HFD-induced gliosis and IEG expression specific to the mice expressing *APOE3* and an increase in Adora2a expression in mice expressing *APOE4*. These changes were apparent without robust changes in an array of other inflammatory genes. This work suggests that initial gliosis and IEGs may perform a novel protective role in response to early stages of obesity. More broadly, this approach of combining strong genetic and environmental factors is necessary for providing insight into personal risks of important CNS impairment and potential for AD development.

## Limitations

While these data identify specific CNS alterations in *APOE3* mice that do not occur in *APOE4*, the study has important limitations. It does not identify any specific mechanisms that lead to the CNS alterations caused by HFD, or the downstream consequences of those alterations. In addition, the conclusions about HFD inducing gliosis in *APOE3* mice are based on small sample numbers, with only three to four animals (of mixed sex) per group. For qRT-PCR analyses of the identified genes, we examined two to three animals per sex per group, and we did not observe any trends suggesting differences by sex. Given the importance of sex in the risk of AD, further studies could be powered to examine the combined effects of *APOE* genotype, and diet, and sex. Finally, while we observed strong differences in gliosis in the HPC, a larger sample size may have allowed us to better test for significant differences in the other areas.

## Data Availability

The datasets used and/or analyzed during the current study are available from the corresponding author on reasonable request.

## Abbreviations

AD: Alzheimer’s disease
AO: Apical oblique
APOE: Apolipoprotein E
BS: Basal shaft
CNS: Central nervous system
CTX: Cortex
DCX: Doublecortin
HFD: High-fat diet
HPC: Hippocampus
IEG: Immediate early gene
LTP: Long-term potentiation
